# Evaluation of Salt‐Tolerant Sweet Potato ( *Ipomoea batatas* (L.) Lam.) Genotypes Using Morphophysiological and Biochemical Markers

**DOI:** 10.1002/pei3.70231

**Published:** 2026-10-25

**Authors:** A. F. M. Shamim Ahsan, Md Shihab Uddine Khan, Nahid Afridi, Sadia Afroz Ritu, Zakaria Alam, Nadira Mokarroma, A. A. M. Mohammad Mustakim, A. H. M. Motiur Rahman Talukder, Md. Anwar Hossain Khan, Akbar Hossain

**Affiliations:** ^1^ Plant Physiology Division Bangladesh Agricultural Research Institute (BARI) Gazipur Bangladesh; ^2^ Department of Genetics and Plant Breeding Bangladesh Agricultural University (BAU) Mymensingh Bangladesh; ^3^ Department of Crop Botany Bangladesh Agricultural University (BAU) Mymensingh Bangladesh; ^4^ Tuber Crops Research Centre Bangladesh Agricultural Research Institute (BARI) Gazipur Bangladesh; ^5^ Centre for Crop and Food Innovation (CCFI) Murdoch University (MU) Murdoch Australia; ^6^ Agronomy Division Bangladesh Wheat and Maize Research Institute Dinajpur Bangladesh

**Keywords:** antioxidant defense, K^+^/Na^+^, ROS, salinity stress, sweet potato

## Abstract

Salinity stress hinders plant physiological processes, resulting in lower crop yields and posing serious challenges to agriculture. Sweet potato ( *Ipomoea batatas* L.) varies among genotypes in terms of salinity tolerance, highlighting the need to select those that maintain development and yield under salinity stress. The morphophysiological and biochemical reactions of four sweet potato genotypes (H‐16‐EJ/10, H‐9.7/12, H‐9.48/11, and CIP‐TCRC‐04) to salinity stress were investigated. Three varying electrical conductivity (EC) values were applied to the sweet potato genotypes: 0.3 as a control, 8, and 12 dS m^−1^. A completely randomized design (CRD) with three replications was used in the experiment. The findings demonstrated that salinity stress (8 and 12 dS m^−1^) decreased the morphological parameters, including vine length, tuber length, branch/plant ratio, marketable tuber weight, tuber diameter, and vine fresh weight. The 2,2‐diphenyl‐1‐picrylhydrazyl (DPPH) (%) was greater for genotypes H‐16‐EJ/10. Compared to the respective controls, an enhancement in salinity led to a reduction in photosynthetic pigment content. Under salinity stress, the concentration of proline increased fivefold, with H‐16‐EJ/10 accumulating the most. With a high K^+^/Na^+^ ratio, H‐16‐EJ/10 and H‐9.7/12 were the most resilient to salinity stress. Genotype H‐16‐EJ/10 was the most salt resistant since it had the highest K^+^/Na^+^ ratio, photosynthetic pigment, antioxidant system activity, and marketable tuber weight and the lowest degree of ROS generation under salinity stress. H‐16‐EJ/10 and H‐9.7/12 could be considered for field trials in saline‐prone areas because of their strong salt tolerance and high tuber yield, and they can also be used for breeding purposes to create new salinity‐tolerant genotypes for sustainable production.

**Abbreviations**
BP: number of branches per plant
CAT: catalase
Chlo A: chlorophyll a
Chlo B: chlorophyll b
CMSI: cell membrane stability index
DAB: 3,3′‐diaminobenzidine
DAP: days after planting
DPPH: 2,2‐diphenyl‐1‐picrylhydrazyl
EC: electrical conductivity
H_2_O_2_
: hydrogen peroxide
MDA: malondialdehyde
MTW: marketable tuber weight
O_2_ **•** ^−^
: superoxide
OH: hydroxyl radical
PCA: principal component analysis
POD: peroxidase
ROS: reactive oxygen species
SOD: superoxide dismutase
TBA: thiobarbituric acid
TCA: trichloroacetic acid
T Chlo: total chlorophyll
TD: tuber diameter
TL: tuber length
VDW: vine dry weight
VFW: vine fresh weight
VL: vine length

## 1 Introduction

Sweet potato ( *Ipomoea batatas* L.) is widely cultivated in tropical, subtropical, and frost‐free temperate temperature zones worldwide. It contains significant amounts of carbohydrates, vitamins A and C, fiber, iron, potassium, dietary protein, and fiber. Cultivars with orange and yellow flesh are enriched in beta‐carotene, which is an antecedent to vitamin A (Motsa et al. 2015). Its high starch and secondary metabolite contents make it suitable for animal nutrition and industrial use (Kim et al. 2013).

Plants in their natural environments frequently encounter multiple stresses that limit development and yield (Mareri et al. 2022). These stresses are generally classified as abiotic (salinity, drought, heat, waterlogging, and heavy metals) or biotic (pathogenic organisms and insects) (Dresselhaus and Hückelhoven 2018). Among abiotic stresses, salinity is among the extremely severe abiotic restraints affecting global agriculture (Rahman et al. 2024). An estimated 1.125 million ha of land worldwide are currently affected through salinity stress (Shahid et al. 2018), with forecasts suggesting that salinization may result in the degradation of around 1.5 million ha of cultivable land each year (Kumar et al. 2023).

Salinity disrupts several morphophysiological, biochemical, and molecular processes in plants (Gupta and Huang 2014). Its adverse effects are primarily driven by ionic imbalance, osmotic disruption, and enhanced oxidative injury, which together compromise plant development and reproductive success. In sweet potato, salinity stress impairs tuber formation by disrupting the ion balance and limiting water absorption, resulting in notable yield losses (Li et al. 2022). Salinity stress also decreases growth, productivity, and tuber quality, particularly under dry and semiarid conditions (Ahanger et al. 2017). Under high salinity, especially at 150 mM NaCl, the vine length, tuber initiation, and overall biomass accumulation of sweet potato significantly decrease (Mondal et al. 2022). Similar declines in both fresh and dry biomass were identified in various crops, such as soybean (Pavli et al. 2021), maize (Zahra et al. 2020) and tomato (Tanveer et al. 2020). In addition to impacting growth, salinity‐induced ionic stress negatively affects plant architecture by decreasing the number of branches (Munns and Tester 2008). Marketable tuber weight is also substantially reduced in saline environments (Mangal et al. 2024), although some studies have reported increased tuber diameter under such stress (Doganay et al. 2023). It impairs photosynthetic performance by lowering the chlorophyll content and intensifying oxidative stress, which in turn affects energy metabolism and plant development.

Ionic balance is disrupted at the cellular level by the accumulation of Na^+^ during salinity stress, causing the efflux of essential cytosolic ions such as K^+^ and Ca^2+^. This ionic imbalance disturbs cellular functions, compromises membrane stability, and inhibits key enzymatic activities necessary for normal metabolic processes (Rahman et al. 2024; Ahanger et al. 2017; Khan et al. 2025). Elevated salt levels also lead to improved levels of MDA, a sign of membrane LPO, and a reduction in the CMSI because of the excessive buildup of soluble salts in sweet potato (Alzahib et al. 2021). It further induces oxidative damage by the overproduction of ROS, including O_2_ **•** ^−^, **•**OH, and H_2_O_2_ (Kesawat et al. 2023). These ROS damage vital cellular structures, affecting activities such as stomatal regulation, photosynthesis, and transpiration. In sweet potato, salinity significantly reduces the photosynthetic pigment content (Acosta‐Motos et al. 2017). To mitigate oxidative damage, plants activate enzymatic defense mechanisms. Salt‐tolerant genotypes often exhibit increased activities of enzymes such as peroxidase, catalase, and superoxide dismutase, which help them detoxify excess ROS (Fujita and Hasanuzzaman 2022). Enhanced POD and CAT action has been informed in sweet potato under salinity stress, although nonenzymatic antioxidants such as 2,2‐diphenyl‐1‐picrylhydrazyl (DPPH) tend to decrease, indicating reduced antioxidant enzymatic potential (Abdalla et al. 2022). Ion homeostasis is also disrupted by salinity, as Na^+^ influx occurs through K^+^ transport pathways, leading to ionic toxicity and oxidative stress. A low K^+^/Na^+^ ratio in plants often indicates salinity stress, whereas tolerant genotypes balance a high K^+^/Na^+^, which is critical for cellular stability and metabolic regulation (Giacopelli et al. 2023).

Salinity stress adversely influences the productivity, tuber formation, and physiological activities of sweet potato by disturbing the ion balance, lowering chlorophyll levels, and intensifying oxidative damage. Nevertheless, it is hypothesized that sweet potato genotypes vary in salt tolerance based on differences in growth, yield, ion homeostasis, chlorophyll pigments, and antioxidant defense mechanisms. The novelty is that the various stress‐response attributes are connected to multivariate analyses to describe genotype‐specific tolerance. Therefore, the objective of this research was to assess the morphophysiological and biochemical responses of selected genotypes under various salinity stresses and identify the tolerant lines based on principal component analysis (PCA) and hierarchical clustering. The findings may support future field validation and provide valuable genetic assets for breeding initiatives aimed at producing salt‐tolerant sweet potato varieties.

## 2 Materials and Methods

### 2.1 Experimental Site and Climate

For identification of salt‐tolerant sweet potato genotypes through morphophysiological and biochemical markers, a pot experiment was conducted in the net house of the Plant Physiology Division of the Bangladesh Agricultural Research Institute (BARI) in Gazipur. The experimental site was positioned in the Gazipur district in the Dhaka division at 23.60′ N latitude and 90.25′ E longitude. The climate was humid, subtropical, and subject to monsoons. The climatological information during crop establishment was obtained from a nearby weather station. The mean temperature fluctuated between 25.5°C and 34.3°C during the crop development period. The mean relative humidity ranged from 80.0% to 84.5%. The rainfall pattern during the investigational time ranged from 0 to 141 mm (Figure 1).

**FIGURE 1:**
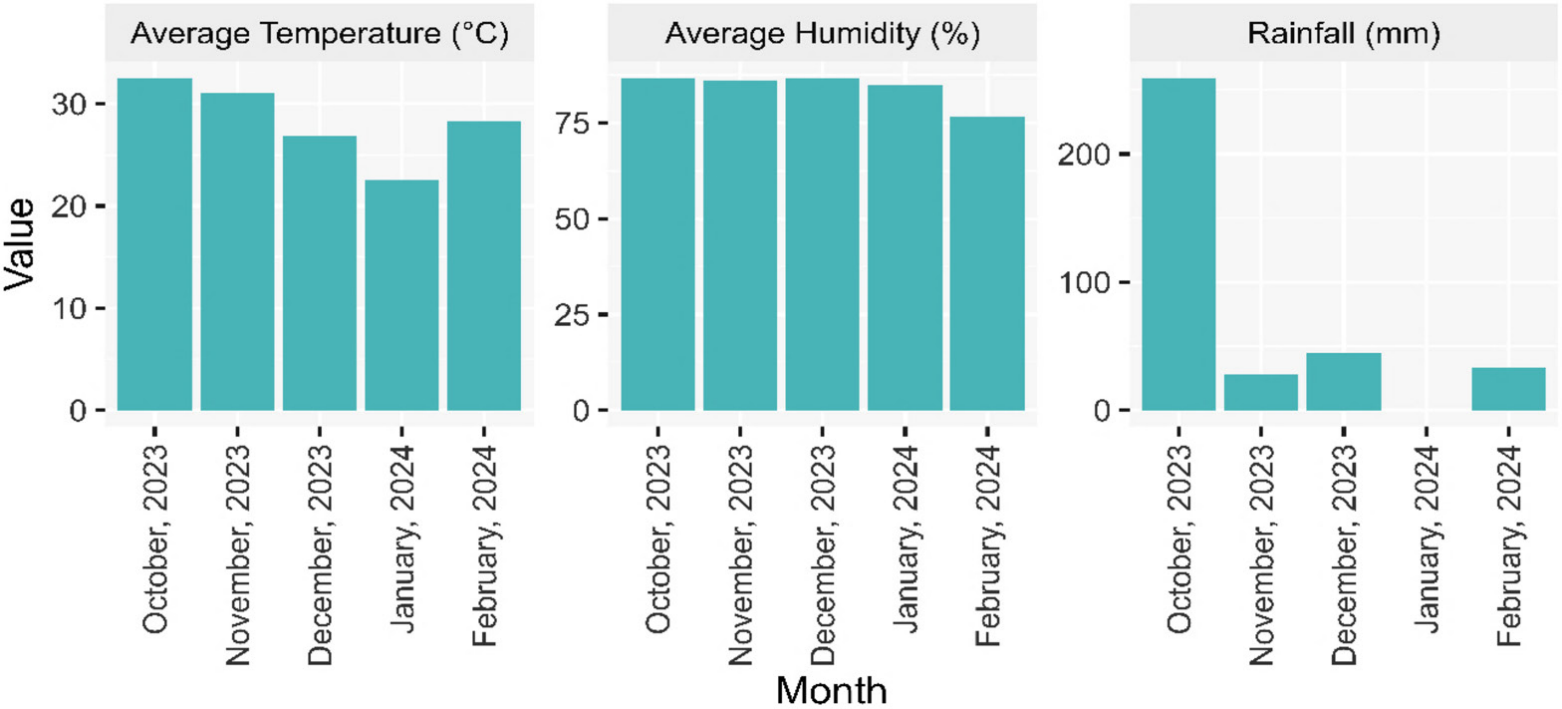
Monthly variations in average temperature (°C), total rainfall (mm), and average humidity (%) at the study location during 2023–2024.

The principal soil type of the soil collected for the pot experiments was terrace soil, and the soil was acidic, clay loam, and belonged to the Chhiata soil series (Agroecological Zone 28) (Shil et al. 2016). Before the pot experiments were initiated, soil samples (0–15 cm depth) were gathered from the field according to the standard procedure, and the standard protocols were used to analyze the physical and chemical characteristics in Tables S1 and S2, respectively.

### 2.2 Experimental Materials and Planting

We used four sweet potato genotypes that were obtained from the Tuber Crops Research Centre (TCRC), BARI, Gazipur. The genotypes evaluated are described in detail in Table 1. All sweet potato vine cuttings were obtained from the TCRC, BARI, Gazipur.

**TABLE 1:** A detailed explanation of the four sweet potato genotypes utilized in this experiment.

Genotypes	Origin	Trade name	Breeding method	Parental information
CIP‐TCRC‐04	Peru	BENJAMIN	Introduction	
H‐16EJ/10	Bangladesh		Half diallel	Female parent: CIP‐441132
H‐9.48/11	Bangladesh		Half diallel	Female parent: BARI Mistialu‐8
H‐9.7/12	Bangladesh		Half diallel	Female parent: BARI Mistialu‐6

### 2.3 Experiments and Design

A completely randomized design (CRD) with three replications was used in the experiment. The study contained three levels of salinity: a control with no salinity stress (0.3 dS m^−1^, using distilled water), 8 dS m^−1^, and 12 dS m^−1^, and four sweet potato genotypes (Table 1). Salinity was imposed at 35 DAP by the addition of a NaCl salt solution. Saline solutions were imposed at increments of 5 dS m^−1^every other day until each container reached the required salinity level over the following 15 days. Using an Hanna HI: 98,331 EC meter, salinity was monitored every 2 days throughout the experiment by inserting the probe directly into the soil of each pot. Salinity levels were maintained by monitoring and adding salt solution when required until maturity. Until maturity, salinity levels were maintained by observing and applying saline solution as needed. The control treatment was adjusted to approximately 0.3 dS m^−1^ using distilled water. Plastic pots (each containing 25 kg of soil) were poured with soil–cow dung (4:1 ratio). All the genotypes were supplied with balanced fertilizer equivalent to 260, 150, 250, 75, 12, and 10 kg/ha of urea, triple superphosphate, muriate of potash, gypsum, zinc sulphate, and boric acid, converted proportionally to the soil weight used in each plastic pot (Ahmmed et al. 2018). Healthy vine cuttings 30 cm in length were planted, with two‐thirds of each cutting inserted into the soil. Three cuttings were established per pot, and after 25 days, two vines were removed from each pot. Tuber harvesting was carried out at 130 DAP, while leaf samples for measurements were collected at 100 DAP.

### 2.4 Measurement of Tuber Yield and Yield‐Related Attributes

Vine length (cm), tuber length (cm), no. of branches per plant, marketable tuber weight per plant (g), tuber diameter (mm), vine fresh weight (g), and vine dry weight (g) were measured following the procedure of Alam et al. (Alam et al. 2023) for each treatment across three pots, and the values were averaged. In accordance with the standards of (United States Department of Agriculture (USDA), Agricultural Marketing Service 2005), marketable sweet potatoes must be firm, clean, and free from defects, with acceptable grades including tubers 7.6–22.9 cm long, 4.45–8.89 cm in diameter, and weighing up to 1.021 kg.

### 2.5 Photosynthetic Pigment Measurements

Chlorophyll and carotenoids in the leaf samples were determined according to the methods of Arnon (Arnon 1949). Leaf samples weighing 500 mg were powdered with 10 mL of 80% acetone at 4°C and centrifuged for 10 min at 2500 rpm. This process was constant till the extract converted colorless. The residue was transferred to an Eppendorf tube, and the volume was increased to 10 mL with 80% acetone and analyzed instantly. Three‐milliliter aliquots of the residue were moved to a quartz cuvette, and a spectrophotometer (UV‐1800; Shimadzu, Japan) was utilized to measure the absorbance at 645, 663, and 480 nm using 80% acetone as a blank. Chlorophyll content was estimated applying the method of Arnon (Arnon 1949) and presented in mg g^−1^ FW.

$$\text{Chlorophyll}\;a\;\left(\mathrm{mg}\;g-1\;\right)=\left\{12.7\;\left(D663\right)-2.69\;\left(D645\right)\right\}\times \frac{V}{1000\times w}$$

$$\text{Chlorophyll}\;b\;\left(\mathrm{mg}\;g-1\right)=\left\{22.9\;\left(D645\right)-4.68\;\left(D663\right)\right\}\times \frac{V\;}{1000\times w}$$

$$\text{Total Chlorophyll}\;\left(\mathrm{mg}\;g-1\right)=\left(\text{Chlorophyll}\;a+\text{Chlorophyll}\;b\right)$$

$$\text{Chlorophyll}\;\frac{a}{b}=\frac{\text{Chlorophyll}\;a}{\text{Chlorophyll}\;b}$$

where *D*, optical density; *V*, final volume of 80% acetone (mL); and *w*, fresh weight of the sample (*g*).

### 2.6 Biochemical Measurements

#### 2.6.1 2,2‐Diphenyl‐1‐Picrylhydrazyl (DPPH)

2,2‐diphenyl‐1‐picrylhydrazyl (DPPH) activity was measured according to previous methods (Tadolini et al. 2000). The reaction method consisted of 50 μL of extract and 2.5 mL of 65 μM DPPH (made from 80% methanol). After incubation for 30 min at 25°C in the dark, the absorbance was determined instantly at 517 nm. DPPH activity was determined by the formula:

DPPH scavenging activity (%) = $\left[1-\frac{\left(A-B\right)}{C}\right]\times 100.$

where *A*: the absorbance values at 517 nm of the reaction mixture containing 50 μL of the sample extractant and 2.5 mL of 65 μM DPPH solution, *B*: the absorbance values at 517 nm of the sample blank containing 50 μL of the sample extractant and 80% methanol and *C*: the absorbance values at 517 nm of the control containing 50 μL of 80% methanol and 2.5 mL of 65 μM DPPH solution.

#### 2.6.2 Determination of Protein

Protein solutions with 10–100 μg of protein dissolved in 0.1 mL were placed in 12 × 100 mm test tubes. An appropriate proper buffer was added to adjust the volume to 0.1 mL for calibration purposes in the test tube. Protein reagent 5 mL was mixed by inversion or vortexing in the test tube. The absorbance at 595 nm was measured in 3 mL cuvettes after 2 min and 1 h before a reagent blank of 0.1 mL buffer and 5 mL protein reagent was used. A standard curve of protein weight vs. absorbance values was generated in order to determine protein content of unknown samples. Every protein sample was determined following Bradford's (Bradford 1976) protocol.

#### 2.6.3 Enzyme Extarction and Essays

Soluble enzymes were extracted from tomato leaf tissue during the anthesis phase by collecting fully mature leaves from five plants/treatment group. Extraction was done according to the method outlined by Rohman et al. (Rohman et al. 2016). This involved mixing tomato leaf tissue (0.5 g) in 1 mL of ice‐cold extraction buffer composed of potassium phosphate (50 mM, pH 7.0), KCl (100 mM), ascorbate (1 mM), β‐mercaptoethanol (10 mM), and 10% glycerol (w/v). These homogenates were then centrifuged for 10 min at a speed of 11,500 × *g* at a temperature of 4°C. The supernatants obtained were used for the estimation of the enzymes and antioxidants.

#### 2.6.4 Catalase (CAT, EC: 1.11.1.6)

Catalase (CAT) activity was assessed by observing the reduction in absorbance at 240 nm for 1 min because of H_2_O_2_ degradation (Hossain et al. 2010). A 50 mM potassium‐phosphate buffer solution (pH 7.0), 15 mM H_2_O_2_, and an enzyme solution in a final volume of 0.7 mL made up the reaction mixture. The enzyme extract was employed to initiate the process, and an extinction coefficient of 39.4 M^−1^ cm^−1^ was utilized to determine the activity.

#### 2.6.5 Peroxidase (POD, EC 1.11.1.7)

Peroxidase (POD) activity was estimated according to the method of Hemeda and Klein (Hemeda and Klein 1990). The reaction mixture contained 25 mM potassium‐phosphate buffer (pH 7.0), 0.05% guaiacol, 10 mM H_2_O_2_, and the enzyme extract. The activity was measured using an extinction value of 26.6 mM^−1^ cm^−1^ and the enhancement in absorbance at 470 nm caused by guaiacol oxidation for 1 min.

#### 2.6.6 Lipid Peroxidation

The level of lipid peroxidation was determined by measuring the level of malondialdehyde, a degradation product of the peroxidized polyunsaturated fatty acid element of the membrane lipid, using TBA as the reactive material following the method of Heath and Packer (Heath and Packer 1968). Describely, the leaf tissue (0.5 g) was homogenized in 3 mL of 5% (w/v) trichloroacetic acid (TCA), and the homogenate was centrifuged at 11,500 × *g* for 10 min. The extract (1 mL) was mixed with 4 mL of TBA (0.5% TBA in 20% TCA).

#### 2.6.7 Histochemical Detection of H_2_O_2_ and O_2_

3,3‐DAB was used as a substrate for visual identification of H_2_O_2_ in the leaves (Thordal‐Christensen et al. 1997). Following collection, the sweet potato leaves were immediately immersed in a 1 mg mL^−1^ DAB solution (pH 3.8) and allowed to incubate for 6 h at 25°C under light. Subsequently, they endured bleaching in boiling ethanol (96%) for a duration of 10 min. This method removed the color from the leaves, except for the brown polymerization component generated by the interaction of DAB and H_2_O_2_.

Superoxide radical levels were determined according to the method of Elstner and Heupel (Elstner and Heupel 1976) with modifications. Leaves (0.3 g) were homogenized in 3 mL of 65 mmol L^−1^ phosphate buffer (pH 7.8) in an ice bath and were then centrifuged at 4°C and 5000 × *g* for 10 min. The supernatants (0.75 mL) were mixed with 0.675 mL of 65 mmol L^−1^ phosphate buffer (pH 7.8) and 0.07 mL of 10 mmol L^−1^ hydroxylamine chlorhydrate and were placed at 25°C. After 20 min, 0.375 mL of 17 mmol L^−1^ sulfanilamide and 0.375 mL of 7 mmol L^−1^ α‐naphthylamine were added, and the mixture was placed at 25°C for another 20 min before it was mixed with 2.25 mL of ether. The absorbance was measured at 530 nm and the O_2_^•−^concentration was calculated from a standard curve of NaNO_2_.

### 2.7 Cell Membrane Stability Index (CMSI)

The CMSI was determined by calculating ion leaching from the leaf tissue into distilled water. The plant material (1.00 g) was added to 20 mL of distilled water in two sets. The conductivity of the first set was measured using a conductivity meter (C1) after it was heated to 25°C for 12 h. The conductivity of the second set was measured (C2) after it was placed in a boiling water bath (100°C) for 10 min. The CMSI was determined from the formula $\text{CMSI}\;\left(\%\right)=\left(1-\frac{C1}{C2}\right)\times 100$ (Ahmed et al. 2013).

### 2.8 Na^+^ and K^+^ Ion Measurements

To measure the Na^+^ and K^+^ levels, sap was collected from freshly picked leaves using a tissue sap extractor. Na^+^ ion (Horiba‐731, Japan) and K^+^ ion (Horiba‐722, Japan) meters were used to measure the ions of Na^+^ and K^+^, respectively (Rohman et al. 2019).

### 2.9 Statistical Analysis

The analysis was completed by using R (v4.5.1) and RStudio software. All measured traits were documented from each biological replicate, and three pots were considered as single replicate observations used for statistical analysis. Tukey's least significant difference (LSD) test, two‐way analysis of variance (ANOVA), and multiple comparisons for mean separation at *p ≤ 0.05*, were used to calculate the significance of the differences among the various groups. The correlation matrix was created using the R package corrplot. The Factoextra, FactomineR, and ggplot2 packages were utilized to create the PCA biplot (Vu 2011). The complexHeatmap package in R program, a two‐way hierarchical clustering heatmap was created using Euclidean distance algorithms (R Studio 2020).

## 3 Results

### 3.1 Effect of Salinity Stress on Sweet Potato Growth and Yield Traits

Salinity stress drastically lowered yield and yield contributing traits across genotypes (Table 2; Table S3). A progressive decline in vine length (VL) was observed with increasing salinity: from 66.66 cm (control) to 36.66 cm (12 dS m^−1^) in CIP‐TCRC‐04, from 139.33 to 118.33 cm in H‐16EJ/10, from 76.00 to 54.00 cm in H‐9.48/11, and from 72.33 to 55.66 cm in H‐9.7/12. In the CIP‐TCRC‐04 genotype, vine length decreased by 23.49% at 8 dS m^−1^ and by 45.00% at 12 dS m^−1^, 11.48% and 15.07% in H‐16EJ/10, 20.18% and 28.95% in H‐9.48/11, 18.90% and 23.05% in H‐9.7/12 respectively. Tuber length (TL) followed a similar trend, decreasing in CIP‐TCRC‐04 (12.66–9.16 cm), H‐16EJ/10 (16.8–14.66 cm), H‐9.48/11 (15.3–12.33 cm), and H‐9.7/12 (15.16–12.83 cm). In the CIP‐TCRC‐04 genotype, tuber length decreased by 3.95% at 8 dS m^−1^ and by 27.65% at 12 dS m^−1^, 5.94% and 12.89% in H‐16EJ/10, 14.38% and 19.78% in H‐9.48/11, 7.65% and 15.37% in H‐9.7/12 respectively. The number of branches per plant (BP) decreased moderately under salinity, from 4 to 3 in CIP‐TCRC‐04, 6.33 to 4.66 in H‐16EJ/10, from 5.33 to 4.66 in H‐9.48/11, and from 5.33 to 4.33 in H‐9.7/12 (Table 2; Table S3).

**TABLE 2:** Interaction effects of genotype and salinity on growth and yield traits of four sweet potatoes.

Genotypes	Salinity (dS m^−1^)	VL (cm)	TL (cm)	BP (No.)	MTW (g)	TD (mm)	VFW (g)	VDW (g)
CIP‐TCRC‐04	Control	66.66 ± 3.5cde	12.66 ± 0.1d	4.00 ± 0.6 cde	384.80 ± 10.5e	29.01 ± 0.7d	146.94 ± 2.9c	20.02 ± 0.7 g
	8	51.00 ± 2.6f	12.16 ± 0.6d	3.33 ± 0.3de	303.03 ± 16.2f	27.36 ± 0.3d	81.16 ± 2de	14.13 ± 0.3 h
	12	36.66 ± 2.7 g	9.16 ± 0.1e	3.00 ± 0.1e	208.58 ± 6.8 h	23.35 ± 1.4e	67.44 ± 3.1e	13.39 ± 1.4 h
HH‐16EJ/10	Control	139.33 ± 8.7a	16.83 ± 0.7a	6.33 ± 0.9a	728.55 ± 48.3a	37.82 ± 0.6b	316.38 ± 10.4a	46.83 ± 0.7a
	8	123.33 ± 7.2b	15.83 ± 0.4ab	5.66 ± 0.3ab	684.68 ± 8.6ab	41.24 ± 1.7a	309.11 ± 3.2a	43.04 ± 0.5b
	12	118.33 ± 4.4b	14.66 ± 1.4bc	4.66 ± 0.3bcd	600.06 ± 32.8c	39.56 ± 0.5ab	284.22 ± 3.3ab	36.79 ± 1d
HH‐9.48/11	Control	76.00 ± 2.5c	15.37 ± 0.5ab	5.33 ± 0.3abc	476.91 ± 14.07d	40.14 ± 0.8ab	267.38 ± 6.8ab	34.82 ± 1.5e
	8	60.66 ± 1.7def	13.16 ± 0.6 cd	5.00 ± 0.9abc	288.23 ± 18.3 fg	32.50 ± 0.4c	132.83 ± 6.4 cd	19.70 ± 1.4 g
	12	54.00 ± 1.7ef	12.33 ± 0.3d	4.66 ± 0.3bcd	230.10 ± 8.3gh	27.98 ± 0.6d	114.72 ± 1.4 cde	18.39 ± 1.2 g
HH‐9.7/12	Control	72.33 ± 4.3 cd	15.16 ± 0.3ab	5.33 ± 0.3abc	732.78 ± 9.5a	41.53 ± 1.8a	297.72 ± 8.1ab	39.34 ± 1c
	8	58.66 ± 1.7ef	14.00 ± 0.7bcd	5.00 ± 0.6abc	691.97 ± 11.6ab	38.94 ± 1.3ab	252.61 ± 10.6b	41.57 ± 1.8b
	12	55.66 ± 2.3ef	12.83 ± 0.4 cd	4.33 ± 0.6bcde	632.09 ± 11bc	39.73 ± 0.8ab	149.61 ± 62.3c	32.10 ± 1.1f

Abbreviations: BP, number of branches per plant; CV, coefficient of variation; LSD, least significant difference; MTW, marketable tuber weight (g/plant); TD, tuber diameter (mm); TL, tuber length (cm); VDW, vine dry weight (g); VFW, vine fresh weight (g); VL, vine length (cm).

The marketable tuber weight (MTW) showed genotype‐dependent responses: in CIP‐TCRC‐04, it decreased substantially (384.80–208.58 g/plant), whereas H‐9.7/12 maintained higher yields (732.78 to 632.09 g/plant) at 12 dS m^−1^. In the CIP‐TCRC‐04 genotype, tuber length decreased by 21.25% at 8 dS m^−1^ and further to 45.80% at 12 dS m^−1^, 6.02% and 17.64% in H‐16EJ/10, 39.56% and 51.75% in H‐9.48/11, 5.57% and 13.74% in H‐9.7/12 respectively. Tuber diameter (TD) decreased in all genotypes except H‐16EJ/10, which increased from 37.82 mm (control) to 41.24 mm (8 dS m^−1^) before declining to 39.56 mm (12 dS m^−1^). Vine biomass consistently decreased at 12 dS m^−1^across all genotypes, with H‐9.48/11 (267.38 to 114.72 g fresh; 34.82 to 18.39 g dry) and CIP‐TCRC‐04 (146.94 to 67.44 g fresh; 20.02 to 13.39 g dry) showing the greatest reductions. However, H‐16EJ/10 maintained the highest biomass, which decreased only slightly from 316.38 to 284.22 g fresh and from 46.83 to 36.79 g dry weight (Table 2; Table S3). In the CIP‐TCRC‐04 genotype, tuber length decreased by 29.42% at 8 dS m^−1^ and further to 33.12% at 12 dS m^−1^, 5.67% and 18.40% in H‐16EJ/10, 43.42% and 47.19% in H‐9.48/11, 8.09% and 21.44% in H‐9.7/12 respectively.

### 3.2 Effects of Salinity Stress on Antioxidant Enzyme Activities and Physiological Responses on Sweet Potato

In the H‐16EJ/10 genotype, the 2,2‐diphenyl‐1‐picrylhydrazyl (DPPH) activity decreased slightly from 80.30% (control) to 71.11% at 8 dS m^−1^and further to 63.55% at 12 dS m^−1^. CAT activity and POD activity increased the most, from 23.57 (control) to 108.49 (12 dS m^−1^) and from 2.13 to 12.53 μmol min^−1^ mg^−1^ protein, respectively (Tables 3 and 4; Table S3). The accumulation of proline was greatest (99.87 mg g^−1^ FW) at 12 dS m^−1^, whereas the increase in MDA was moderate (2.96 to 9.29 nmol^−1^g^−1^FW). Among all the genotypes, the CMSI was highest at 49.58 at 12 dS m^−1^. Na^+^ increased from 250 (control) to 600 ppm (12 dS m^−1^), whereas K^+^ decreased from 3200 to 3000 ppm, and the K^+^/Na^+^ ratio decreased from 12.80 to 5.00. The concentration of chlorophyll a (Chlo A) decreased from 0.91 (control) to 0.51 (12 dS m^−1^), that of chlorophyll b (Chlo B) decreased from 1.55 to 0.99, and that of total chlorophyll (T Chlo) decreased from 2.46 to 1.50 mg g^−1^ FW (Tables 3 and 4; Table S3).

**TABLE 3:** Interaction effects of genotype and salinity on antioxidant enzyme activities and physiological responses of four sweet potatoes.

Genotypes	Salinity level (dS m^−1^)	DPPH (%)	CAT (μmol min^−1^ mg^−1^ protein)	POD (μmol min^−1^ mg^−1^ protein)	MDA (nmol^−1^g^−1^FW)	Proline (mg g^−1^ FW)	CMSI (%)
CIP‐TCRC‐04	Control	88.53 ± 0.3a	26.38 ± 0.02j	1.64 ± 0.01 h	3.67 ± 0.09 h	18.66 ± 0.1 g	74.24 ± 0.04c
	8	49.74 ± 0.1 g	33.06 ± 0.01 h	3.76 ± 0.02f	11.33 ± 0.1c	47.60 ± 0.1f	47.48 ± 0.1 h
	12	23.29 ± 0.5 j	59.66 ± 0.3f	6.38 ± 0.2d	16.78 ± 0.09a	62.75 ± 3.5d	35.78 ± 0.2 k
H‐16EJ/10	Control	80.30 ± 0.1c	23.57 ± 0.03 k	2.13 ± 0.04gh	2.96 ± 0.02i	17.43 ± 0.2 g	78.34 ± 0.01a
	8	71.11 ± 0.4e	76.50 ± 0.3d	7.34 ± 0.1b	6.50 ± 0.1f	68.39 ± 0.2c	61.34 ± 0.03d
	12	63.55 ± 0.1f	108.49 ± 0.1a	12.53 ± 0.2a	9.29 ± 0.1e	99.87 ± 0.4a	49.58 ± 0.2 g
H‐9.48/11	Control	82.54 ± 0.3b	27.33 ± 0.04i	2.38 ± 0.0 2 g	3.83 ± 0.04 h	17.74 ± 0.1 g	76.40 ± 0.1b
	8	63.46 ± 0.1f	51.41 ± 0.1 g	5.38 ± 0.1e	10.35 ± 0.1d	49.80 ± 0.3f	53.73 ± 0.2f
	12	47.67 ± 0.1 h	84.50 ± 0.1c	6.55 ± 0.2 cd	14.45 ± 0.1b	61.24 ± 0.1de	41.40 ± 0.1j
H‐9.7/12	Control	78.03 ± 0.4d	23.46 ± 0.1 k	2.04 ± 0.06gh	3.04 ± 0.02i	18.62 ± 0.2 g	74.55 ± 0.1c
	8	50.18 ± 0.1 g	63.22 ± 0.2e	7.03 ± 0.08bc	5.43 ± 0.2 g	59.75 ± 0.3e	57.29 ± 0.2e
	12	35.34 ± 0.1i	100.52 ± 0.1b	12.40 ± 0.2a	10.03 ± 0.07d	90.34 ± 0.1b	45.34 ± 0.1i

Abbreviations: CAT, catalase (μmol min^−1^ mg^−1^ protein); CMSI, cell membrane stability index (%); CV, coefficient of variation; DPPH, 2,2‐diphenyl‐1‐picrylhydrazyl (%); LSD, least significant difference; MDA, malondialdehyde (nmol^−1^ g^−1^ FW); POD, peroxidase (μmol min^−1^ mg^−1^ protein).

**TABLE 4:** Interaction effects of genotype and salinity on photosynthetic pigments and ionic homeostasis of four sweet potatoes.

Genotype	Salinity level (dS m^−1^)	Chlo A (mg g^−1^ FW)	Chlo B (mg g^−1^ FW)	T chlo (mg g^−1^ FW)	Na^+^ (ppm)	K^+^ (ppm)	K^+^/Na^+^
CIP‐TCRC‐04	Control	0.96 ± 0.02a	1.48 ± 0.05bc	2.44 ± 0.01a	316.66 ± 8.8 h	3216.66 ± 72.6b	10.16 ± 0.09e
	8	0.46 ± 0.02 h	1.01 ± 0.02 g	1.47 ± 0.01f	1150.00 ± 17.3c	2400.00 ± 57.7f	2.08 ± 0.01j
	12	0.32 ± 0.01j	0.78 ± 0.02i	1.10 ± 0.01 h	1500.00 ± 23.1a	1916.66 ± 44.1 h	1.27 ± 0.01 k
H‐16EJ/10	Control	0.91 ± 0.02c	1.55 ± 0.06a	2.46 ± 0.01a	250.00 ± 5.7i	3200.00 ± 57.7b	12.80 ± 0.06c
	8	0.65 ± 0.02d	1.21 ± 0.03d	1.86 ± 0.01c	240.00 ± 11.5ij	2566.66 ± 88.1e	10.70 ± 0.1d
	12	0.51 ± 0.02 g	0.99 ± 0.03 g	1.50 ± 0.02f	600.00 ± 11.5f	3000.00 ± 57.7c	5.00 ± 0.01 g
H‐9.48/11	Control	0.93 ± 0.02b	1.52 ± 0.03ab	2.45 ± 0.03a	140.00 ± 5.7 k	3000.00 ± 115.4c	21.43 ± 0.06a
	8	0.53 ± 0.02f	1.07 ± 0.05f	1.60 ± 0.01e	1000.00 ± 17.3d	2816.66 ± 44.1d	2.81 ± 0.01i
	12	0.32 ± 0.01j	0.74 ± 0.01i	1.06 ± 0.01 h	1200.00 ± 17.3b	2600.00 ± 57.7e	2.16 ± 0.01j
H‐9.7/12	Control	0.92 ± 0.02b	1.45 ± 0.05c	2.37 ± 0.01b	230.00 ± 5.7j	3300.00 ± 57.7a	14.35 ± 0.1b
	8	0.59 ± 0.02e	1.13 ± 0.03e	1.72 ± 0.01d	380.00 ± 5.7 g	2333.33 ± 33.3f	6.14 ± 0.04f
	12	0.40 ± 0.02i	0.83 ± 0.02 h	1.24 ± 0.01 g	650.00 ± 11.5e	2000.00 ± 57.7 g	3.07 ± 0.03 h

Abbreviations: Chlo A, chlorophyll a (mg g^−1^ FW); Chlo B, chlorophyll b (mg g^−1^ FW); CV, coefficient of variation; K^+^, Potassium ion (ppm); LSD, least significant difference; Na^+^, Sodium ion (ppm); T Chlo, total chlorophyll (mg g^−1^ FW).

The antioxidant capacity of the CIP‐TCRC‐04 decreased, with the 2,2‐diphenyl‐1‐picrylhydrazyl (DPPH) content decreasing from 88.53% (control) to 23.29% (12 dS m^−1^). CAT activity and POD activity increased from 26.38 to 59.66 and from 1.64 to 6.38 μmol/min/mg P, respectively. The level of proline accumulation was 62.75 mg g^−1^ FW at 12 dS m^−1^, whereas the level of MDA enhanced sharply from 3.67 to 16.78 nmol^−1^g^−1^FW. T Chlo declined sharply (2.44 to 1.10 mg g^−1^ FW), with Chlo A decreasing from 0.96 to 0.32 and Chlo B decreasing from 1.48 to 0.78 mg g^−1^ FW. However, it maintained the lowest CMSI of 35.78 at 12 dS m^−1^. Na^+^ increased from 316.66 ppm (control) to 1500 ppm (12 dS m^−1^), K^+^ decreased (3216.66 to 1916.66 ppm), and the K^+^/Na^+^ ratio reduced sharply (10.16–1.27) (Tables 3 and 4; Table S3).

Genotypes H‐9.48/11 and H‐9.7/12 displayed intermediate ion imbalances between H‐16EJ/10 and CIP‐TCRC‐04. In H‐9.48/11, Na^+^ increased to 1200 ppm, and in H‐9.7/12, to 650 ppm. The K^+^/Na^+^ ratios decreased to 2.16 and 3.07, respectively. At 12 dS m^−1^, the proline concentration was 61.24 mg g^−1^ FW in H‐9.48/11 and 90.34 mg g^−1^ FW in H‐9.7/12. The MDA concentration increased to 14.45 nmol^−1^g^−1^FW in H‐9.48/11 and 10.03 in H‐9.7/12. Their DPPH activities also moderately decreased to 47.67% for H‐9.48/11 and 35.34% for H‐9.7/12. In H‐9.48/11, the CAT activity and POD activity reached 84.50 and 6.55 μmol min^−1^ mg^−1^ protein, respectively, whereas in H‐9.7/12, the values increased to 100.52 and 12.40 μmol min^−1^ mg^−1^ protein, respectively. Chlo A decreased from 0.93 to 0.32 mg g^−1^ FW in H‐9.48/11 and from 0.92 to 0.40 in H‐9.7/12, whereas Chlo B decreased from 1.52 to 0.74 mg g^−1^ FW in H‐9.48/11 and from 1.45 to 0.83 in H‐9.7/12. T Chlo declined to 1.06 mg g^−1^ FW in H‐9.48/11 and 1.24 in H‐9.7/12 at 12 dS m^−1^ (Tables 3 and 4; Table S3).

### 3.3 Histochemical Analysis of O_2_ •^−^ and H_2_O_2_

In H‐16EJ/10, the staining for O_2_ **•** ^−^ and H_2_O_2_ remained consistently light at both 8 and 12 dS m^−1^, with a slight enhancement in intensity observed at higher salinity. Conversely, CIP‐TCRC‐04 exhibited much darker staining that intensified as the salinity increased, particularly at 12 dS m^−1^ (Figure 2).

**FIGURE 2:**
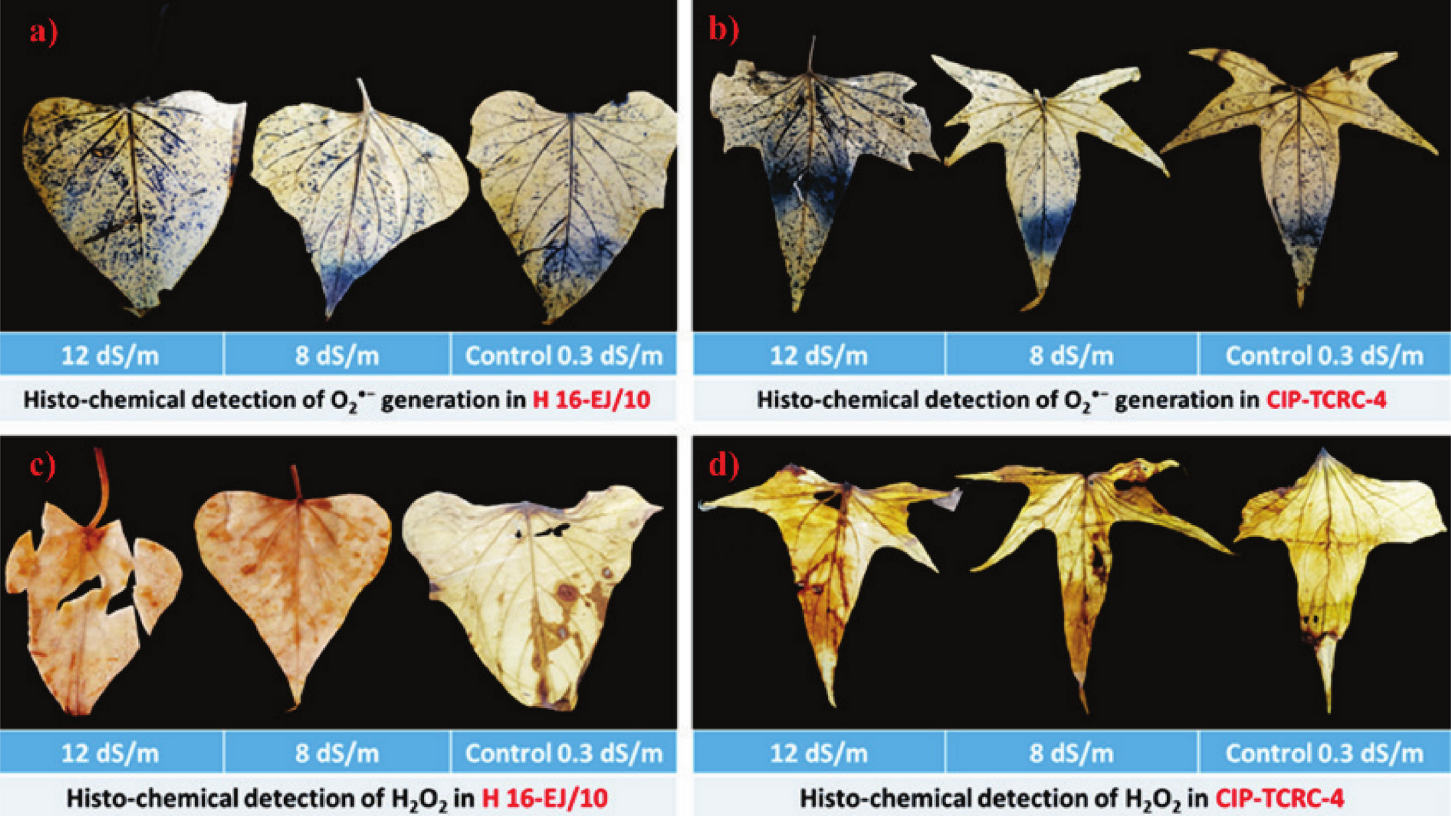
Histochemical analysis of ROS in sweet potato leaves under various salinity levels. Superoxide radicals (O_2_ **•** ^−^) were detected in genotypes H‐16EJ/10 (a) and CIP‐TCRC‐04 (b) Hydrogen peroxide (H_2_O_2_) was detected in genotypes H‐16EJ/10 (c) and CIP‐TCRC‐04 (d) Histochemical detection was used to visualize the localization of ROS in the plant tissues. Darker colors correlated with higher ROS production.

### 3.4 Correlations of Traits for Different Sweet Potato Genotypes Under Different Salinity Stresses

The correlation matrix revealed significant relationships under salinity stress (Figure 3). Traits such as VL, VFW, VDW, MTW, TD, TL, and BP showed strong positive correlations and were negatively associated with Na^+^ accumulation. The yield‐related traits (e.g., MTW, TL, and TD) were positively associated with the photosynthetic content (chlorophyll), K^+^/Na^+^ ratio, and the CMSI. Moreover, antioxidant activities, including CAT, POD, and proline, and 2,2‐diphenyl‐1‐picrylhydrazyl (DPPH), were positively correlated. Conversely, Na^+^ was negatively connected with both K^+^ and the K^+^/Na^+^ (Figure 3).

**FIGURE 3:**
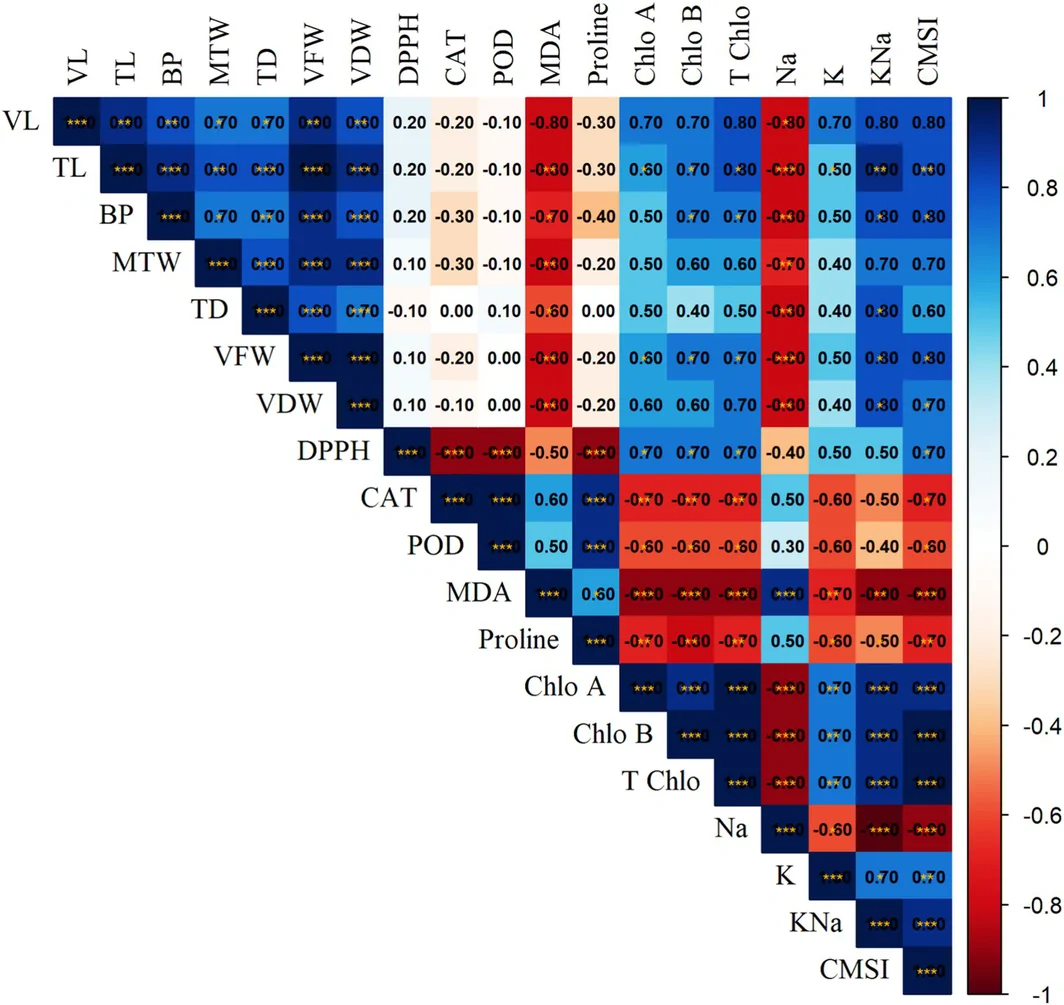
A correlation matrix of seven evaluated tuber yield‐contributing attributes and 13 biochemical traits under salinity stress conditions was constructed based on Pearson's correlation. Red and blue indicate negative and positive correlation. The greater the color concentration is, the greater the association coefficient. Asterisks confirm a substantial correlation among attributes. *, **, and ***, indicate 5%, 1% and 0.1% significance. Nonsignificant (*p* > 0.05) correlations are described by blank blocks. BP, Number of branches per plant; CAT, Catalase (μmol min⁻¹ mg⁻¹ protein); Chlo A, Chlorophyll a (mg g⁻¹ FW); Chlo A/B, Chlorophyll a/Chlorophyll b; Chlo B, Chlorophyll b (mg g⁻¹ FW); CMSI, Cell membrane stability index; DPPH, 2,2‐diphenyl‐1‐picrylhydrazyl; K, K⁺ (ppm); KNa, K⁺/Na⁺ ratio; MDA, Malondialdehyde (nmol⁻¹ g⁻¹ FW); MTW, Marketable tuber weight (g/plant); Na, Na⁺ (ppm); POD, Peroxidase (μmol min⁻¹ mg⁻¹ protein); TD, Tuber diameter (mm); TL, Tuber length (cm); T Chlo, Total chlorophyll (mg g⁻¹ FW); VDW, Vine dry weight; VL, Vine length (cm); VFW, Vine fresh weight (g).

### 3.5 Relationships Between the Measured Traits of Sweet Potato Genotypes and Treatment Combinations

PCA clarified 88.6% of the overall variance, with Dim1 and Dim2 calculating for 64.4% and 24.2%, respectively (Figure 4a). Trait associations with principal components indicated that Dim1 was the most influential (Figure 4a). The biplot revealed distinct genotype‐treatment responses under salinity. V2T1, V2T2, and V2T3 were associated with higher POD, CAT, Chlo A, Chlo B, T Chlo, K^+^, the K^+^/Na^+^ratio, and yield‐related traits (MTW, TD, VFW, VDW, BP, and TL), indicating superior performance across treatments. V4T2 was located near the origin but showed moderate alignment with yield‐related and physiological traits. V1T3 and V3T3 were located on the negative side of Dim1 and were linked to increased Na and MDA levels, indicating stress sensitivity. V1T1 had a weak correlation with favorable traits and was located in the descending‐right quadrant area. Hence, the H‐16EJ/10 genotype consistently performed better across treatments (Figure 4b).

**FIGURE 4:**
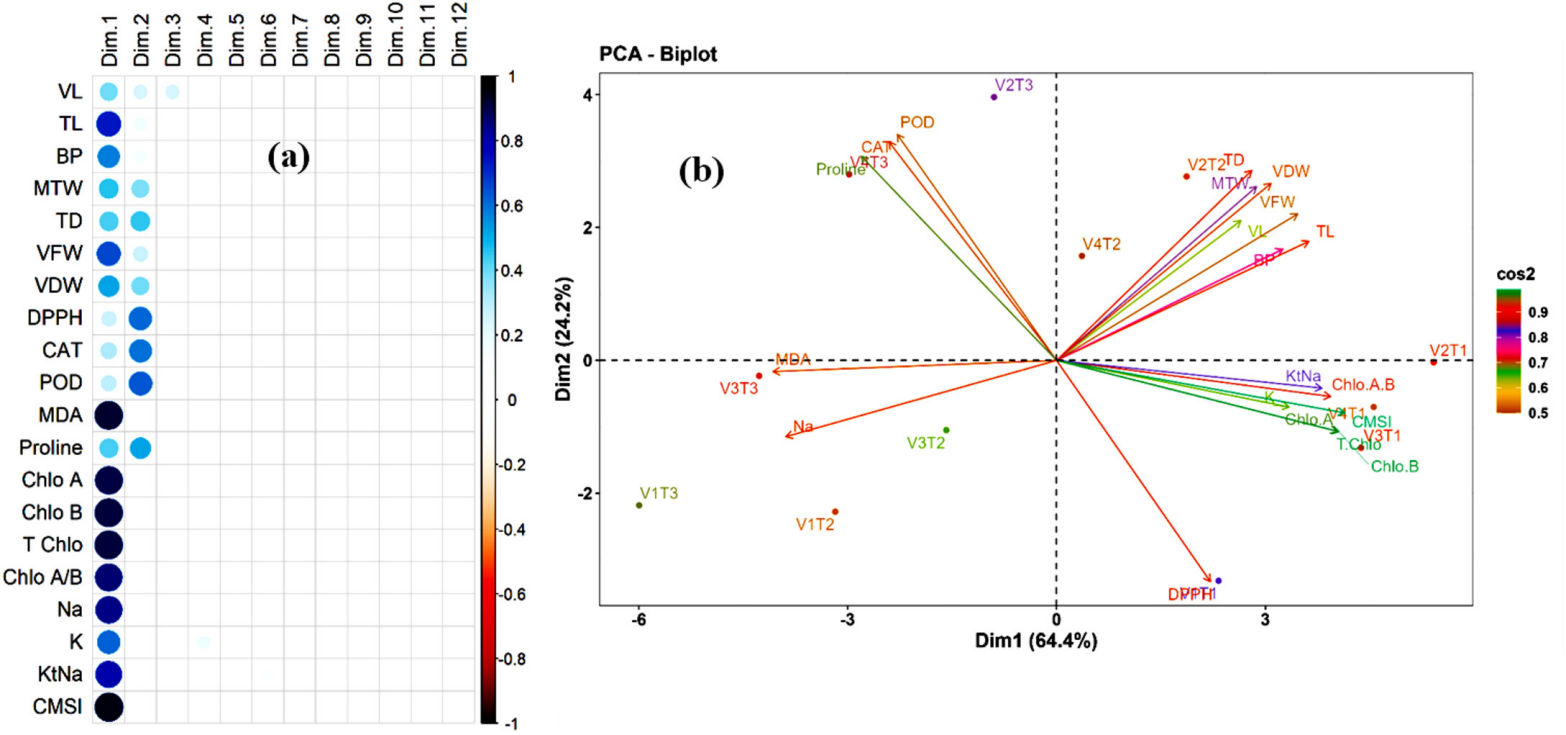
The contribution patterns of attributes to the significant principal components (Dim 1 and Dim 2) (a) followed by a visualization of the relationships among treatments and traits using PCA (b). BP, Number of branches per plant; CAT, Catalase (μmol min⁻¹ mg⁻¹ protein); Chlo A, Chlorophyll a (mg g⁻¹ FW); Chlo A/B, Chlorophyll a/Chlorophyll b; Chlo B, Chlorophyll b (mg g⁻¹ FW); CMSI, Cell membrane stability index; K, K⁺ (ppm); KNa, K⁺/Na⁺ ratio; MDA, Malondialdehyde (nmol⁻¹ g⁻¹ FW); MTW, Marketable tuber weight (g/plant); Na, Na⁺ (ppm); POD, Peroxidase (μmol min⁻¹ mg⁻¹ protein); T1, Control; T2, 8 dS m⁻¹; T3, 12 dS m⁻¹; TD, Tuber diameter (mm); T Chlo, Total chlorophyll (mg g⁻¹ FW); V1, CIP‐TCRC‐04; V2, H‐16EJ/10; V3, H‐9.48/11; V4, H‐9.7/12; VDW, Vine dry weight; VL, Vine length (cm); VFW, Vine fresh weight (g).

### 3.6 Grouping Treatment Combinations for Sweet Potato Traits Using Heatmaps and Hierarchical Clustering Analysis

Heatmaps and hierarchical clustering analysis were used to categorize the genotypes into three distinct clusters based on their morpho‐physiological and biochemical responses under salinity stress conditions (Figure 5). Cluster A included V2T3, V4T3, V2T2, and V4T2, with V2T3 and V4T3 demonstrating the highest level of salt tolerance, strong antioxidant activity (CAT, POD), increased proline accumulation, and superior yield traits (such as VDW, MTW, and TD). However, V2T3 and V4T3 were slightly negatively correlated with 2,2‐diphenyl‐1‐picrylhydrazyl (DPPH) activity. Cluster B included V1T3, V3T3, V1T2, and V3T2, with V1T3 having the highest Na^+^ and MDA levels. Cluster C (V1T1, V2T1, V3T1, and V4T1) represented the control group with no salinity stress (Figure 5).

**FIGURE 5:**
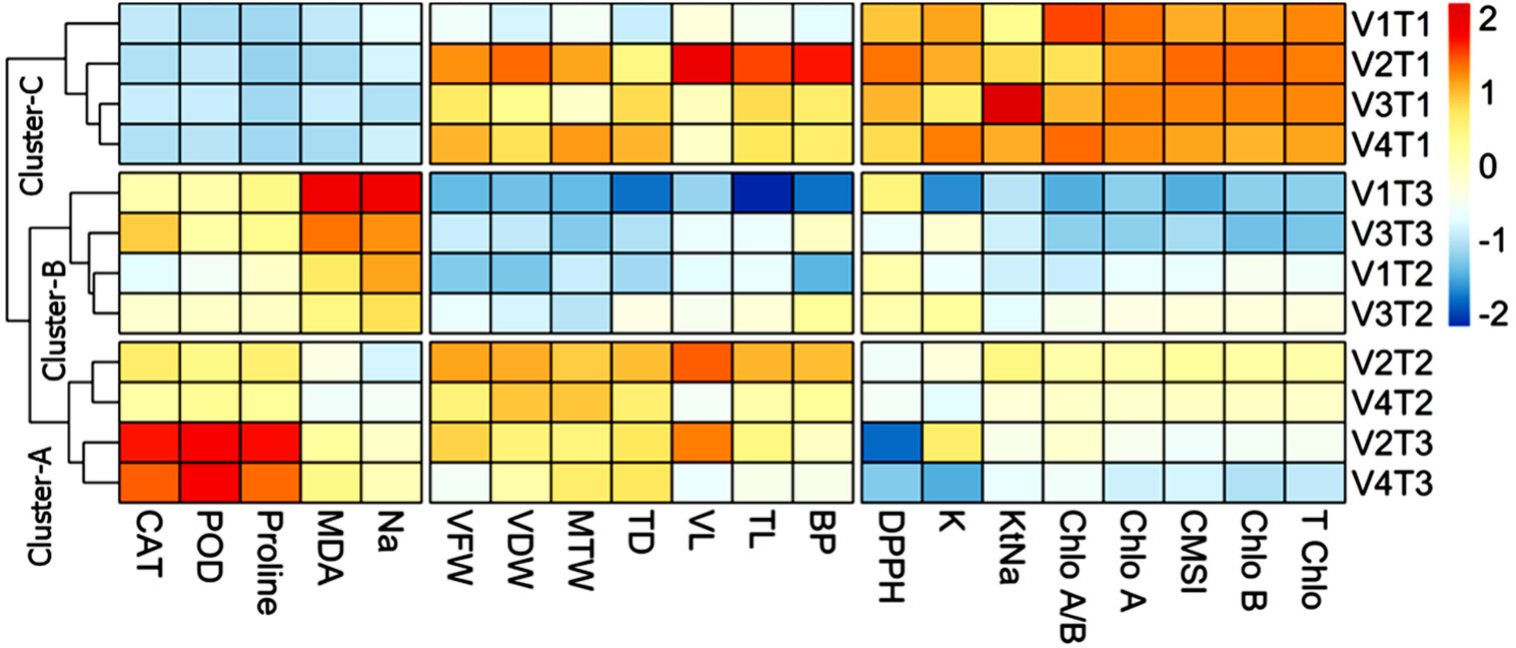
Hierarchical clustering and heatmaps for the 12 treatment combinations and 20 diverse traits under salinity stress environments. Contributors are identical to a comparative scale of −2 to +2 for the heatmap. Numerous attributes are expressed by unalike columns, where treatment combinations are denoted by unique rows of the heatmap. Treatment combinations and traits are both clustered into three groups (Cluster A, B and C). The concentration of the treatment combination–attribute correlation is determined by managing discrete colors and their strengths. Salt sensitivity is shown in deep blue, and salt tolerance is displayed in dark red. BP, Number of branches per plant; CAT, catalase (μmol min⁻¹ mg⁻¹ protein); Chlo A, chlorophyll a (mg g⁻¹ FW); Chlo A/B, chlorophyll a/chlorophyll b; Chlo B, chlorophyll b (mg g⁻¹ FW); CMSI, cell membrane stability index; K, K⁺ (ppm); KtNa, K⁺/Na⁺ ratio; MDA, malondialdehyde (nmol⁻¹ g⁻¹ FW); MTW, marketable tuber weight (g/plant); Na, Na⁺ (ppm); POD, peroxidase (μmol min⁻¹ mg⁻¹ protein); T1, control; T2, 8 dS m⁻¹; T3, 12 dS m⁻¹; TD, tuber diameter (mm); T Chlo, total chlorophyll (mg g⁻¹ FW); V1, CIP‐TCRC‐04; V2, H‐16EJ/10; V3, H‐9.48/11; V4, H‐9.7/12; VDW, vine dry weight; VL, vine length (cm); VFW, vine fresh weight (g).

## 4 Discussion

### 4.1 Effects of Salinity on Sweet Potato Growth, Yield, and Genotypic Responses

Salinity stress at 8 and 12 dS m−1significantly decreased sweet potato growth and yield by adversely impacting VL, TL, BP, TD, VFW, VDW, and MTW, according to previous studies (Sanwal et al. 2022). These reductions were attributed mainly to osmotic stress and Na^+^induced ionic imbalance. Genotypic differences significantly influence yield‐related trait performance under saline conditions (Alam et al. 2023). Among the tested genotypes, CIP‐TCRC‐04 was the most sensitive, showing pronounced reductions at 12 dS m^−1^. H‐9.48/11 exhibited decreases in VFW, VDW, and TL, whereas BP remained relatively stable. In contrast, H‐16EJ/10 maintained VFW and VDW with only minor reductions in MTW, VL, and TL and increased TD at 8 dS m^−1^, which is consistent with the findings of Doganay et al. (Doganay et al. 2023). Additionally, H‐9.7/12 produced the highest MTW under saline conditions, indicating moderate salt tolerance.

### 4.2 Antioxidant Enzyme Responses and Oxidative Stress

Under salinity stress, the activity of the antioxidant enzymes CAT and POD increased across all genotypes, with H‐16EJ/10 exhibiting the highest levels at 12 dS m^−1^, indicating enhanced ROS detoxification. Elevated ROS typically induce lipid peroxidation and membrane linkage, as exhibited by enhanced malondialdehyde levels (Fujita and Hasanuzzaman 2022; Jafari et al. 2022). CIP‐TCRC‐04 displayed the highest MDA content and lowest CMSI, indicating higher lipid peroxidation and reduced membrane stability, whereas H‐16EJ/10 maintained the highest CMSI, demonstrating superior membrane stability, which is consistent with previous observations (Awaad 2023). Genotypes H‐9.48/11 and H‐9.7/12 showed intermediate responses, with H‐9.7/12 better preserving membrane integrity under high salinity.

### 4.3 Nonenzymatic Antioxidant Capacity (2,2‐Diphenyl‐1‐Picrylhydrazyl (DPPH) Activity)

2,2‐diphenyl‐1‐picrylhydrazyl (DPPH) activity, which is indicative of nonenzymatic antioxidant capacity, decreased with increasing salinity in all the genotypes (Abdalla et al. 2022). Notably, H‐16EJ/10 decreased the least, maintaining high scavenging activity at 12 dS m^−1^, suggesting strong antioxidant defenses. In contrast, the abundance of CIP‐TCRC‐04 markedly decreased, indicating greater susceptibility to oxidative stress. These findings align with observations by Alvan et al. (Alvan et al. 2025).

### 4.4 Proline Accumulation and Osmoprotection

The accumulation of proline, an osmolyte involved in protein and membrane stabilization and osmotic balance, increased significantly in each genotype's responses to salinity stress (de Freitas et al. 2018). H‐16EJ/10 resulted in the greatest increase in proline levels, indicating strong osmoprotection, whereas H‐9.48/11 resulted in the lowest increase, potentially linked to its reduced salt tolerance. However, proline accumulation can also reflect cellular stress and damage under severe conditions (Zulfiqar and Ashraf 2023). Similar to our findings, Sanwal et al. (Sanwal et al. 2022) reported higher proline accumulation in salt‐tolerant genotypes, contributing to reduced ROS damage.

### 4.5 Chlorophyll Content and Photosynthetic Performance

The chlorophyll content of chlorophyll a, chlorophyll b, and total chlorophyll decreased under salinity stress, which may be interlinked with inhibited biosynthesis and enhanced degradation by ROS (Hosseini et al. 2021). Compared with chlorophyll b, chlorophyll a exhibited greater sensitivity, indicating reduced photosynthetic efficiency (Sayyad‐Amin et al. 2016). The greatest chlorophyll loss was observed in CIP‐TCRC‐04 and H‐9.48/11, while H‐9.7/12 maintained the greatest amount of chlorophyll loss, and H‐16EJ/10 was moderately retained, which correlated with improved photosynthetic performance under stress.

### 4.6 Ionic Balance Under Salinity Stress

Salinity stress disrupted ionic balance through increasing Na^+^ uptake and reducing K^+^ levels, thereby dipping the K^+^/Na^+^ ratio (Wang et al. 2017). Enzyme activity depends on stating a high K^+^/Na^+^ ratio and cellular function under salinity stress, as it reflects a plant's ability to exclude or compartmentalize toxic Na^+^ while essential K^+^ is retained (Guo et al. 2020). Munns and Tester (Munns and Tester 2008) reported that this ratio in salt‐tolerant genotypes results from active ion transport and homeostatic regulation rather than from growth dilution effects. In this study, CIP‐TCRC‐04 showed the greatest disruption of ionic balance, indicating weak Na^+^ exclusion or compartmentalization, whereas H‐16EJ/10 maintained the highest K^+^/Na^+^ ratio under salinity stress, demonstrating effective ion regulation associated with salt tolerance, same as the observations reported by Yu et al. (Yu et al. 2016). Nevertheless, the observed changes in K^+^/Na^+^ ratio are regarded as differences in ionic homeostasis rather than confirmation of particular regulatory mechanisms, since the current findings did not specifically assess ion transport pathways, transporter function, or cellular Na^+^ compartmentation.

### 4.7 ROS Accumulation Under Salinity Stress

Elevated levels of reactive oxygen species, including O_2_ **•** ^−^ and H_2_O_2_, induced by salinity cause cellular damage and inhibit plant growth (Hasanuzzaman et al. 2021). Histochemical staining revealed comparatively lighter ROS accumulation in H‐16EJ/10 at both 8 and 12 dS m^−1^ demonstrating efficient ROS scavenging and oxidative stress mitigation (Lei et al. 2023). Conversely, intense staining of CIP‐TCRC‐04 occurred at 12 dS m^−1^, indicating greater ROS accumulation and exposure to oxidative injury. Gill et al. (Gill et al. 2019) reported similar findings.

### 4.8 Trait Correlations Under Salinity Stress

The correlation matrix indicated positive associations among yield attributes and negative correlations with Na^+^ accumulation, reflecting the sodium‐induced disruption of osmotic balance, nutrient accumulation, and yield under salinity stress conditions (Almeida et al. 2017). The positive relationship between the K^+^/Na^+^ ratio and chlorophyll content highlights the role of potassium in maintaining photosynthesis under high salinity (Imtiaz et al. 2023). Increased antioxidant activity, proline accumulation, and 2,2‐diphenyl‐1‐picrylhydrazyl (DPPH) activity indicated coordinated biochemical defenses that enhanced salt tolerance, which is consistent with earlier studies (Cai and Gao 2020).

### 4.9 Multivariate Analyses of Genotypic Variation in Salt Tolerance

PCA revealed that H‐16EJ/10 was associated with high POD activity, high CAT activity, high chlorophyll retention, and favorable yield traits under salinity stress, confirming its superior tolerance mechanisms. These findings support those of Ahmed et al. (Ahmed et al. 2024), who identified antioxidant upregulation as key to salinity resilience. The maintenance of K^+^/Na^+^ homeostasis further emphasized its role as a critical salt tolerance marker (Hussain et al. 2021). In contrast, CIP‐TCRC‐04 clustered with traits of oxidative damage, including elevated Na^+^ and MDA levels, indicating ion toxicity and membrane injury (Siddiqui et al. 2017). The clear separation between H‐16EJ/10 and CIP‐TCRC‐04 along PCA dimension 1 highlights their contrasting adaptive strategies.

Cluster and heatmap analyses revealed that H‐16EJ/10 and H‐9.7/12 performed best under both 8 and 12 dS m^−1^, exhibiting high CAT and POD activities along with sustained chlorophyll and proline levels. These traits contribute to their physiological resilience and stable yields (Saxena et al. 2019). Although 2,2‐diphenyl‐1‐picrylhydrazyl (DPPH) activity slightly decreased, this likely reflects only in vitro antioxidant capacity, as in vivo ROS detoxification depends mainly on enzymatic antioxidants (Azeem et al. 2023). Conversely, CIP‐TCRC‐04 and H‐9.48/11 accumulated elevated Na^+^ and MDA levels, indicating that increased ionic toxicity and oxidative stress led to reduced yields (Faried et al. 2022).

## 5 Conclusion

The results of this research showed that the evaluated genotypes displayed notable differences in morphology, yield‐related traits, and biochemical responses. Among them, H‐16‐EJ/10 demonstrated the greatest degree of salinity tolerance, which was supported by its superior yield attributes, greater chlorophyll content, stronger antioxidant activity, and efficient ionic regulation. Its ability to maintain sodium levels below toxic thresholds indicates an enhanced capacity to cope with salinity stress. Elevated antioxidant activity, higher potassium concentrations, and increased levels of proline and 2,2‐diphenyl‐1‐picrylhydrazyl (DPPH) activity further contribute to its tolerance. In contrast, CIP‐TCRC‐04 was identified as the most sensitive genotype, characterized by excessive sodium accumulation and a relatively low chlorophyll content. To improve salinity tolerance in sweet potato, selecting genotypes with well‐regulated ionic balance and strong antioxidant defense systems is important. Since these results were obtained under pot conditions, further evaluation of the H‐16‐EJ/10 genotype could be considered for multi‐location field validation under salinity‐prone areas in Bangladesh. Specifically, pot experiments restrict soil volume and root growth, may modify water–salt dynamics, and may not accurately represent the heterogeneous salt distributions common in field soils. These variables can affect the expression of genes related to genotypic salt tolerance.

## Funding

The study was financially supported by Bangladesh Agricultural Research Institute, Gazipur 1701, Bangladesh.

## Ethics Statement

The sweet potato genotypes used in this study were developed through hybridization from cultivated genotypes, and their use complied with institutional and national guidelines for plant research. No specific permits were needed.

## Consent

All the authors agree to the publication of this study.

## Conflicts of Interest

The authors declare no conflicts of interest.

## Supporting information


**Table S1:** Physical properties of preliminary soil of the pot experiment.
**Table S2:** Chemical properties of preliminary soil of the pot experiment.
**Table S3:** Analysis of variance of different traits analyzed in four sweet potato genotypes under salinity stress in pot experiment.

## Data Availability

All data are available in Supporting Information and in tables and figures.
