# Supplementary material for: Evaluation of Salt‐Tolerant Sweet Potato ( Ipomoea batatas (L.) Lam.) Genotypes Using Morphophysiological and Biochemical Markers
Source: Plant Environ Interact. 2026 Oct 25;7(5):e70231. doi: 10.1002/pei3.70231 (PMC13696905; doi:10.1002/pei3.70231)
Supplement: Supplementary file 1 [file PEI3-7-e70231-s001.docx]

**Table S1.** Physical properties of preliminary soil of the pot experiment

| **Physical properties** | **Soil test value** | **Interpretation** |
| --- | --- | --- |
| Sand (%) | 32.2 | [87] |
| Silt (%) | 37.2 |  |
| Clay (%) | 30.6 |  |
| Textural class | Clay loam | [88] |
| Field capacity moisture content (%) | 26.5% | - |
| Wilting point moisture content (%) | 16% | - |
| Particle density (g/cc) | 2.50 | [88] |
| Bulk density (g/cc) | 1.43 | [88] |
| Porosity (%) | - | - |
| Soil temperature (^0^C) | 18.8 | - |

**Table S2.** Chemical properties of preliminary soil of the pot experiment

| **Soil depth (0-15 cm)** | **pH** | **EC** | **Organic**  **matter** | **Total N** | **Ca** | **Mg** | **K** | **P** | **S** | **B** | **Zn** |
| --- | --- | --- | --- | --- | --- | --- | --- | --- | --- | --- | --- |
|  |  | **dS/m** | **%** | | **Meq/100 g** | | | **ppm** | | | |
| Initial soil value | 6.1 | 0.09 | 1.20 | 0.066 | 4.66 | 1.13 | 0.12 | 11.0 | 13.5 | 0.17 | 0.90 |
| Critical level | 5.5-6.5 | - | - | 0.12 | 2.0 | 0.5 | 0.12 | 7 | 10 | 0.20 | 0.60 |
| Interpretation [36] | Slightly acidic | Non saline | Low | Very low | Optimum | Medium | Low | Medium | Low | Low | Low |

EC=Electric conductivity, N=Nitrogen, Ca=Calcium, Mg=Magnesium, K=Potassium, P=Phosphorus, S=Sulphur, B=Boron, Zn= Zinc

**Table S3.** Analysis of variance different analysed traits in four sweet potato genotypes under salinity stress in pot experiment

| Traits | Source of variation | | | LSD (5%) | F-value | P-value |
| --- | --- | --- | --- | --- | --- | --- |
|  | **Genotype (G)** | **Treatments (T)** | **G × T** |  |  |  |
| Vine length (VL) | *** | *** | * | 12.94 | 0.06 | 0.94 |
| Tuber length (TL) | ** | *** | * | 1.94 | 0.90 | 0.42 |
| Number of branches per plant (BP) | *** | * | ** | 1.51 | 1.08 | 0.36 |
| Marketable tuber weight (MTW) | *** | *** | ** | 60.24 | 0.58 | 0.57 |
| Tuber diameter (TD) | *** | *** | *** | 3.17 | 0.89 | 0.42 |
| Vine fresh weight (VFW) | *** | *** | ** | 54.41 | 1.53 | 0.24 |
| Vine dry weight (VDW) | ** | *** | *** | 1.83 | 32.09 | 3.01 |
| 2,2-Diphenyl-1-picrylhydrazyl (DPPH) | *** | *** | *** | 0.59 | 6.40 | 0.01 |
| Catalase (CAT) | *** | *** | *** | 0.51 | 4.12 | 0.67 |
| Peroxidase (POD) |  |  |  |  |  |  |
| Malondialdehyde (MDA) | *** | *** | *** | 0.43 | 0.10 | 0.92 |
| Proline | *** | *** | *** | 2.95 | 2.22 | 0.13 |
| Cell membrane stability index (CMSI) | *** | *** | *** | 0.54 | 0.18 | 0.84 |
| Chlorophyll a (Chlo A) | *** | *** | *** | 0.02 | 204.12 | 6.25 |
| Chlorophyll b (Chlo B) | *** | *** | *** | 0.04 | 61.54 | 9.75 |
| Total chlorophyll (T Chlo) | *** | *** | *** | 0.05 | 96.78 | 1.25 |
| Na^+^ | *** | *** | *** | 17.16 | 48.67 | 8.35 |
| K^+^ | *** | *** | *** | 69.52 | 80.46 | 7.62 |
| K^+^/Na^+^ ratio | ** | * | ** | 0.19 | 2.96 | 0.07 |
